# Low molecular weight components of pollen alter bronchial epithelial barrier functions

**DOI:** 10.1080/15476286.2015.1062316

**Published:** 2015-07-15

**Authors:** Cornelia Blume, Emily J Swindle, Stefanie Gilles, Claudia Traidl-Hoffmann, Donna E Davies

**Affiliations:** 1 Brooke Laboratory; Clinical and Experimental Sciences; Faculty of Medicine; University of Southampton; University Hospital Southampton; Southampton, UK; 2 Institute of Environmental Medicine; UNIKA-T; Technische Universität Munich; Munich, Germany; 3 CK CARE; Christine Kühne Center for Allergy Research and Education; Davos, Switzerland; 4 Southampton NIHR Respiratory Biomedical Research Unit; University Hospital Southampton; Southampton, UK

**Keywords:** bronchial epithelial barrier, polarized mediator release, pollen, pollen-associated lipid mediator, tight junctions

## Abstract

The bronchial epithelium plays a key role in providing a protective barrier against many environmental substances of anthropogenic or natural origin which enter the lungs during breathing. Appropriate responses to these agents are critical for regulation of tissue homeostasis, while inappropriate responses may contribute to disease pathogenesis. Here, we compared epithelial barrier responses to different pollen species, characterized the active pollen components and the signaling pathways leading to epithelial activation. Polarized bronchial cells were exposed to extracts of timothy grass (*Phleum pratense*), ragweed (*Ambrosia artemisifolia*), mugwort (*Artemisia vulgaris*), birch (*Betula alba*) and pine (*Pinus sylvestris*) pollens. All pollen species caused a decrease in ionic permeability as monitored trans-epithelial electrical resistance (TER) and induced polarized release of mediators analyzed by ELISA, with grass pollen showing the highest activity. Ultrafiltration showed that the responses were due to components <3kDa. However, lipid mediators, including phytoprostane E1, had no effect on TER, and caused only modest induction of mediator release. Reverse-phase chromatography separated 2 active fractions: the most hydrophilic maximally affected cytokine release whereas the other only affected TER. Inhibitor studies revealed that JNK played a more dominant role in regulation of barrier permeability in response to grass pollen exposure, whereas ERK and p38 controlled cytokine release. Adenosine and the flavonoid isorhamnetin present in grass pollen contributed to the overall effect on airway epithelial barrier responses. In conclusion, bronchial epithelial barrier functions are differentially affected by several low molecular weight components released by pollen. Furthermore, ionic permeability and innate cytokine production are differentially regulated.

## Introduction

The airway epithelium is the first site of contact for inhaled environmental materials (noxious gases, anthropogenic and natural particulates, including pathogens) and its barrier properties are crucial for regulation of innate barrier immunity and control of tissue homeostasis. Epithelial barrier functions can be divided into 3 types: physical, chemical and immunological.^1,2^ The physical barrier arises from the propensity of epithelial cells to elaborate a range of cell-cell contacts including tight junctions, adherens junctions and desmosomes that link with the cytoskeleton to form a robust sheet-like structure. The epithelial barrier is selectively permeable due to the presence of sub-apical tight junctions, which are formed by transmembrane and intracellular proteins that link to the actin cytoskeleton. These junctions form around the whole perimeter of each cell, forming a continuous belt-like structure that regulates paracellular permeability to ions and macromolecules, as well as the polarity of the epithelium.^3^ Located below the tight junctions are the adherens junctions that also link to the actin cytoskeleton, and desmosomes which link to intermediate filaments. These two types of junctions do not directly seal the space between epithelial cells, but they are critical for providing the adhesive force to ensure the integrity of the cell layer. Many proteins are involved in the regulation of the junctional complexes and these not only play a role in adhesion, but also in transcriptional regulation to modulate cell function.^4^

The chemical barrier of the pseudostratified airway epithelium is elaborated by the goblet cells and submucosal glands that secrete mucus, a fluid containing hydrated gel-forming mucins and a range of host defense and cytoprotective molecules, including anti-microbial molecules, anti-proteases and antioxidants. This mucus traps and inactivates inhaled particles and facilitates their clearance via the mucociliary escalator. In addition to these physical and chemical barriers, airway epithelial cells play a key role in immune surveillance, contributing to innate immunity by the secretion of immuno-stimulatory and modulatory mediators including cytokines, chemokines, growth factors and lipid mediators.^2^ Critical to function of this immunological barrier are the tight junctions that determine apical-basolateral polarity and vectorial cytokine release.

It has been hypothesized that abnormalities in airway epithelial barrier properties are central to many lung diseases, including bronchial asthma.^5^ Genome wide association studies have shown that many of the asthma susceptibility genes are expressed in the airway epithelium.^6^ Furthermore, epidemiological studies suggest that environmental interactions with the airway epithelium in early life, e.g. pollution and viral infections, increase susceptibility for development of asthma.^7,8^ Exposure to airborne allergen carriers such as pollen has also been linked to an increased risk of asthma^9^ and, airborne pollen is able to trigger asthma exacerbations.^10^

After inhalation, pollen grains become hydrated on the aqueous surface of the airway epithelium and, within a short period release various substances including allergens and many low molecular weight substances.^11^ It has been shown that pollen associated lipid mediators, like phytoprostane E1 (PPE_1_), have immuno-modulatory properties on dendritic cells favoring TH_2_-mediated T cell responses.^12^ The relevance of lipids in the regulation of epithelial barrier function is supported by a study showing that lipoxin A_4_ increases the physical barrier properties of airway epithelial cells.^13^ Furthermore, peroxisome proliferator-activated receptor (PPAR) γ is a known receptor of many lipid mediators including prostaglandins^14^ and it has been shown that PPARγ is involved in the regulation of the tight junction complex.^15^ Previously we showed that grass pollen extracts do not disrupt the physical barrier of primary human bronchial epithelial cells (PBECs) differentiated at the air-liquid interface, however they did stimulate polarized release of mediators; furthermore, we observed a different pattern of mediator responses in differentiated PBECs derived from patients with severe asthma.^16^ The aim of this study was to compare epithelial barrier responses to different pollen species, characterize the active pollen components and the signaling pathways leading to epithelial activation.

## Results

### Effect of pollen on bronchial epithelial barrier functions

**Figure 1.**
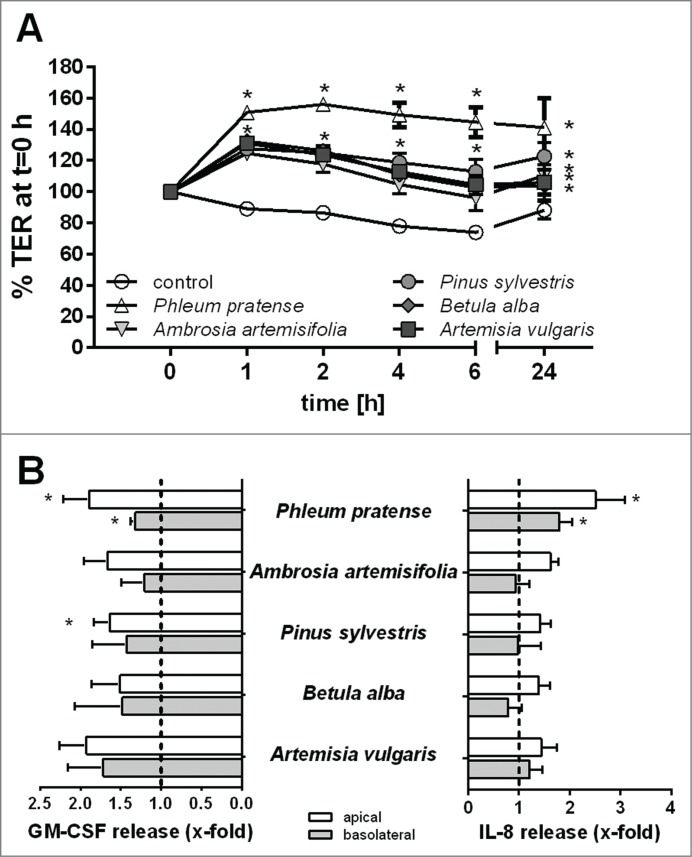
Effect of pollen extract derived from different species on the barrier functions of bronchial epithelial cells. Polarized 16HBE cells were exposed to an extract equivalent of 5 mg/ml pollen. (**A**) Trans-epithelial resistance (TER) measured over time. TER is normalized to t = 0 h directly after stimulation (n = 5). (**B**) Apical and basolateral release of GM-CSF and IL-8 induced by pollen after 24 h measured by ELISA (n = 5–7). Mean ±SEM; *: p ≤ 0.05 compared to untreated control (2-way ANOVA Bonferroni's multiple comparison).

First, we characterized the effect of pollen extracts from different plant species on the barrier functions of polarized 16HBE cells. The physical barrier properties were monitored by measuring the transepithelial electrical resistance (TER) and the immunological barrier by analyzing polarized release of mediators by ELISA. Stimulation of polarized 16HBE cells with extracts of pollen from timothy grass (*Phleum pratense*), ragweed (*Ambrosia artemisifolia*), pine (*Pinus sylvestris*), birch (*Betula alba*) or mugwort (*Artemisia vulgaris*) resulted in a significant increase in TER (**Fig. 1A**). The maximum increase was observed between 1–2 h after stimulation and this was maintained over 24h. Interestingly, an increase in TER was detected even though protease activity could be detected in the pollen extract, with *Ambrosia* showing the highest protease activity (**Fig. S1**), but still causing a significant increase in TER across the epithelial layer. Furthermore, all pollen species induced polarized release of GM-CSF mainly to the apical compartment (**Fig. 1B**) with the effects of *Phleum* (timothy grass) and *Pinus* pollen extracts reaching statistical significance. IL-8 release was also observed, however this only reached significance for *Phleum* pollen extracts which stimulated IL-8 release into both apical and basolateral compartments (**Fig. 1B**).

**Figure 2.**
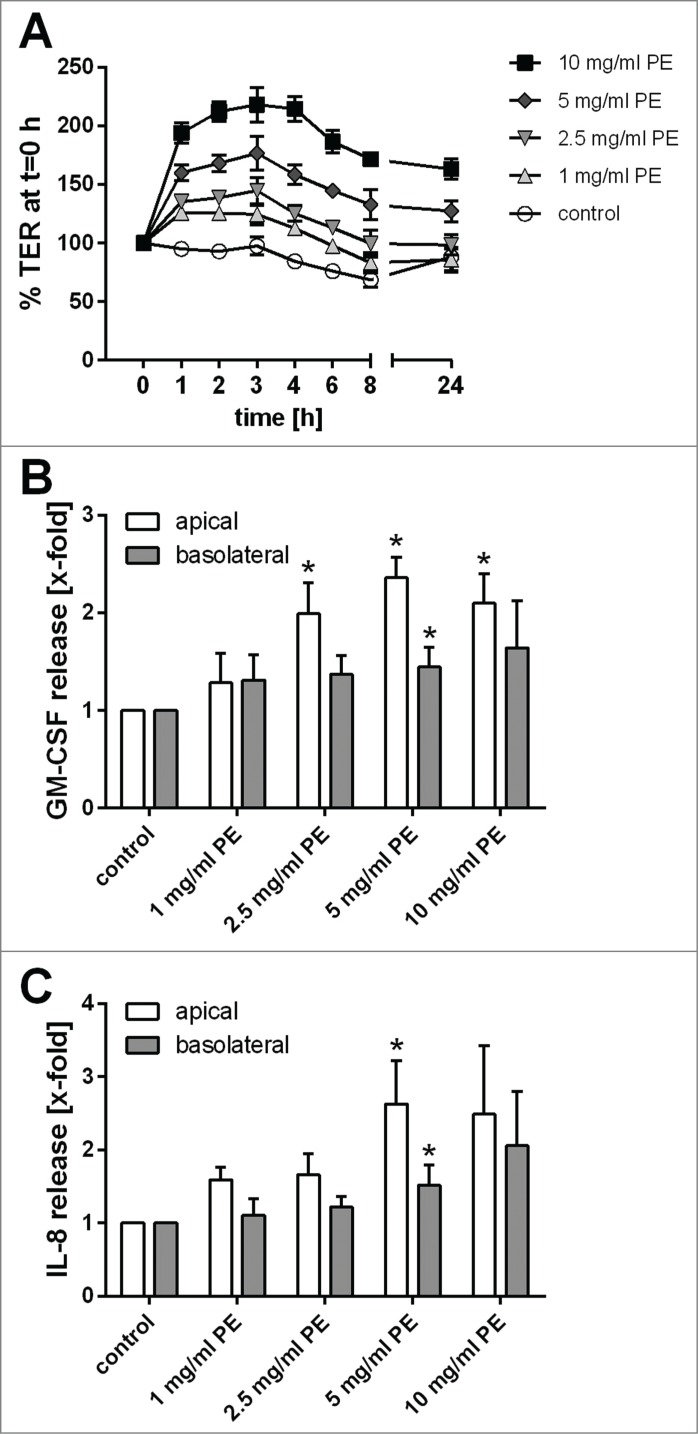
Concentration-dependent effect of grass pollen extract (PE) on bronchial epithelial barrier functions. (**A**) Trans-epithelial resistance (TER) is normalized to t = 0 h (n = 5–8). Release of GM-CSF (**B**) and IL-8 (**C**) induced by grass pollen analyzed by ELISA (n = 5–8). Mean ±SEM; *: p ≤ 0.05 compared to untreated control (Mann-Whitney).

**Figure 3.**
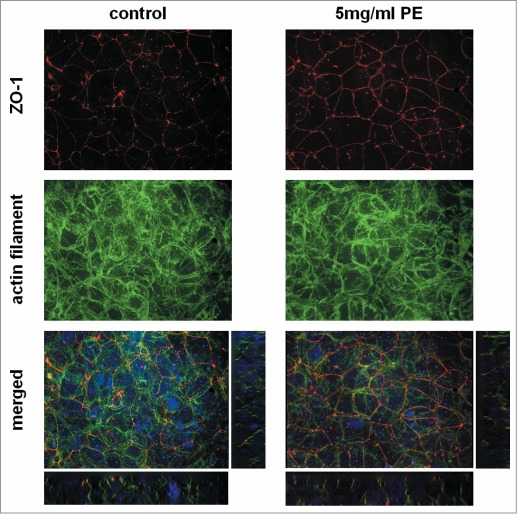
Cellular localization of the tight junction protein ZO-1 in bronchial epithelial cells after exposure to grass pollen extract (PE). Polarized 16HBEs were exposed for 24 h to an equivalent of 5mg/ml pollen and stained by immunofluorescence for ZO-1 (red) and the actin filament (green). Nuclei are shown in blue. Z-projections and orthogonal views are shown. Images are representative of 3 independent experiments.

Since pollen extract from timothy grass showed the highest activity, we further characterized its effects on bronchial epithelial barrier functions. The increase in TER induced by grass pollen extract was concentration-dependent (**Fig. 2A**) and the release of GM-CSF (**Fig. 2B**) and, to a lesser extent, IL-8 (**Fig. 2C**) also showed a concentration-dependent relationship. Since an increase in TER is mostly likely correlated with a tightening of the physical barrier mediated by tight junction proteins, we analyzed the cellular distribution of ZO-1 and actin filaments by fluorescence microscopy. As shown in **Figure 3**, ZO-1 was exclusively localized at the apical side of the polarized 16HBE cell layer and treatment with pollen resulted in more distinct ZO-1 staining, with all apical cells being surrounded by a continuous ring of ZO-1. Furthermore, staining of the actin filaments was less diffuse in the treated cells implying increased organization of the actin cytoskeleton in response to pollen extract.

### Pollen extract induces a polarized release of mediators from epithelial cells

**Figure 4.**
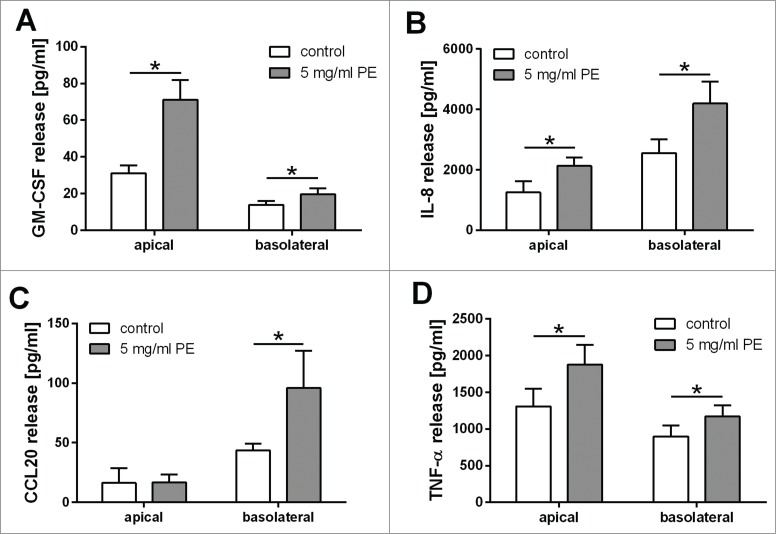
Polarized release of IL-8, GM-CSF, CCL20 and TNF-α induced by grass pollen extract (PE). Polarized 16HBEs were exposed for 24 h to an equivalent of 5 mg/ml pollen and the apical and basolateral release of GM-CSF (**A**), IL-8 (**B**), TNF-α (**C**) and CCL20 (**D**) were analyzed by ELISA (n = 9; CCL20 apical n = 4)). Mean ±SEM; *: p ≤ 0.05 compared to untreated control (Wilcoxon).

Since the integrity of the physical barrier was not disrupted by exposure to pollen extracts, we analyzed the vectorial release of several immunological mediators into the apical and basolateral compartments of our cell culture model. This showed that grass pollen extract stimulated polarized 16HBE cells to release GM-CSF, CCL20, IL-8 and TNF-α. As shown in Figure 4, the release of GM-CSF and CCL20 is highly polarized. GM-CSF was mainly released to the apical compartment, whereas CCL20 was released to the basolateral compartment after treatment with grass pollen extract. In contrast, the release of IL-8 after pollen treatment was increased similarly in both apical and basolateral compartments. The concentration of released TNF-α in untreated cells was comparable in the apical and basolateral compartments. After treatment with pollen extract, the release of TNF-α was increased mainly in the apical compartment.

### Characterization of active compounds in grass pollen extract

**Figure 5.**
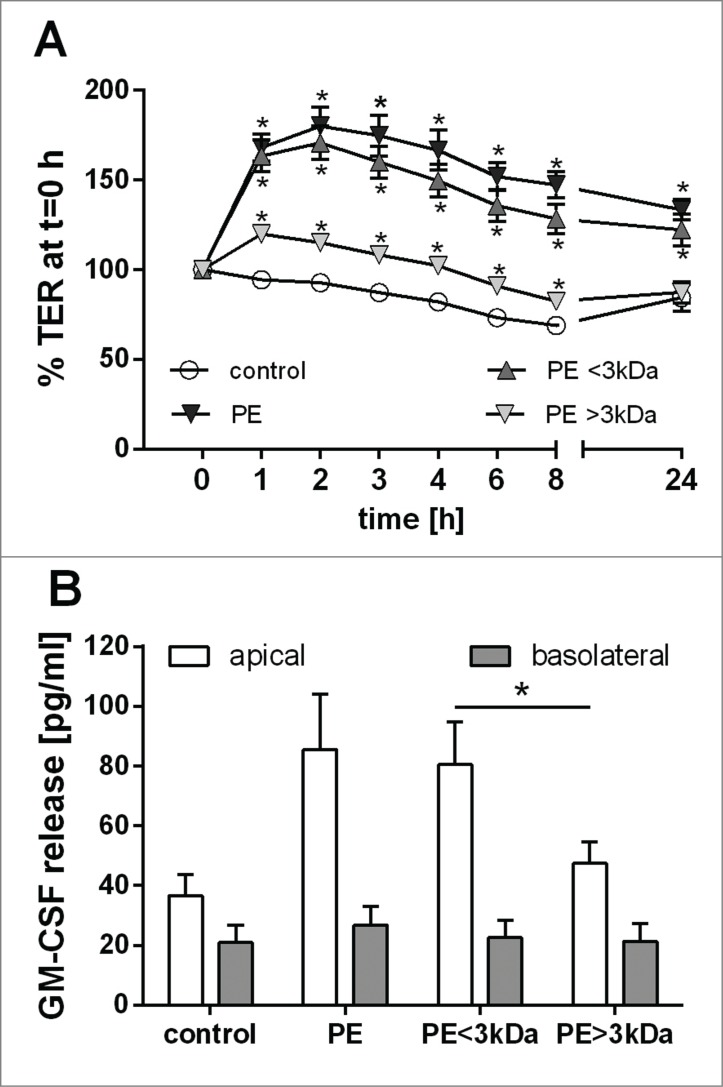
Low molecular weight substances of grass pollen alter bronchial epithelial barrier functions. Grass pollen extract (PE) was separated by ultrafiltration into fractions <3kDa and >3kDa. Polarized 16HBEs were stimulated apically with an equivalent of 5 mg/ml pollen. (**A**) Trans-epithelial resistance (TER) over time (n = 7). TER is normalized to t=0 h. Mean ±SEM; *: p ≤ 0.05 compared to untreated control (Wilcoxon). (**B**) Apical and basolateral release of GM-CSF over 24 h analyzed by ELISA (n = 8). Mean ±SEM; *: p ≤ 0.05 (Wilcoxon).

In order to characterize the active compound(s) in the pollen extract that were capable of affecting the epithelial barriers, grass pollen extract was separated into fractions lower and higher than 3kDa by ultrafiltration. As shown in **Fig. 5A**, only the <3kDa fraction caused an increase in the TER which was comparable to total pollen extract. Similarly, the <3kDa fraction also caused apical release of GM-CSF, which was not observed in the >3kDa fraction (**Fig. 5B**). These data suggest that substances in the pollen of a molecular weight lower than 3kDa are responsible for the effects observed on the epithelial barrier.

Previously, it has been shown that low molecular weight substances isolated from pollen can modulate immune responses mediated by dendritic cells, and the pollen-associated lipid mediator phytoprostane E1 (PPE_1_) was identified as active compound.^17^ Therefore we determined the effect of PPE_1_ on bronchial epithelial barrier functions. Since barrier improving effects of lipoxin A4 have been reported previously, we also included this lipid mediator into our studies. Similarly, as peroxisome proliferator activated receptor gamma (PPARγ) plays a critical role in the control of epithelial barrier functions^15^ and can be activated by natural products, as well as fatty acids and prostanoids, we tested the involvement of PPARγ in pollen-mediated barrier functions. However, treatment of polarized 16HBE cells with PPE_1_ or lipoxin A_4_ did not alter the ionic permeability (Suppl. **Fig. 2A, B**). Similarly treatment with the PPARγ agonist ciglitazone did not affect the TER (Suppl. **Fig. 2C**); Furthermore, the PPARγ antagonist T0070907 caused only a modest reduction of the pollen-induced increase in TER which failed to reach statistical significance.

We also assessed the effects of the lipid mediators on cytokine production. PPE_1_ weakly modulated the immunological barrier properties by showing a trend for a concentration-dependent increase in apical release of GM-CSF (Suppl. **Fig. 3A**). However, the observed mediator release induced by PPE_1_ was below the level induced by total pollen extract. Furthermore, release of IL-8 and TNF-α was not significantly affected by any PPE_1_ concentration (**Fig. S3B, C**). Similarly, the effects of lipoxin A_4_ on apical GM-CSF release and basolateral IL-8 release were weak and did not reach statistical significance (Suppl. **Fig. 4A, B**). Interestingly, the PPARγ antagonist T0070907 significantly enhanced apical and basolateral release of IL-8, and showed a similar trend for apical release of GM-CSF. This was modestly increased by co-exposure to grass pollen (**Fig. S4A,B**). The effects of ciglitazone on GM-CSF or IL-8 release (**Fig. S4A,B**) were not significant.

Since our data suggested that lipid mediators were not the main class of substances released from pollen that altered epithelial barrier functions, we additionally analyzed the effect of the flavonoid isorhamnetin on bronchial epithelial barrier function. As shown in **Supplemental Figure 5**, isorhamnetin did not alter the physical barrier properties but showed a significant increased apical release of GM-CSF and IL-8, almost comparable to that of pollen extract.

**Figure 6.**
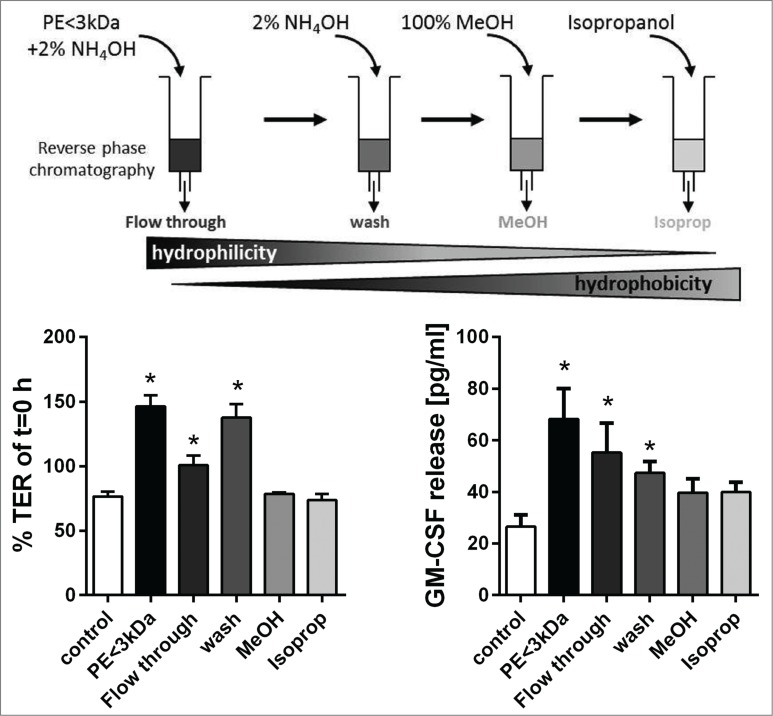
Characterization of substances released by grass pollen that alter the barrier functions of bronchial epithelial cells. The low molecular weight fraction (<3kDa) of pollen extract (PE) was separated by chromatography and resulting fractions were used to stimulate polarized 16HBE cells apically. Trans-epithelial resistance (TER) was measured 2 h after stimulation and normalized to t = 0 h (n = 5). After 24 h apical release of GM-CSF was analyzed by ELISA (n = 4–9). Mean ±SEM; *: p ≤ 0.05 compared to untreated control (Mann-Whitney).

As a series of known lipid mediators did not mimic the effects of grass pollen, we further characterized the active components of grass pollen, by separating the <3kDa fraction of pollen by reverse phase chromatography to isolate substances according to their hydrophobicity. As shown in **Figure 6**, the unbound, hydrophilic components (flow through fraction) caused only a slight increase in TER in the first 2h whereas the weakly hydrophobic wash fraction caused a similar effect on the TER compared to the <3kDa fraction before separation. In contrast, apical release of GM-CSF was most strongly induced by the flow through fraction, but a smaller stimulatory effect was also observed in the wash fraction.

In summary, separation of the <3kDa fraction of grass pollen extract by reverse phase chromatography indicated that the substances responsible for inducing mediator release and altering the ionic barrier properties are mainly hydrophilic molecules, since little binding to the reverse phase cartridge was observed. Furthermore, the data suggest that the ionic and immunological barrier properties are differentially regulated, since the TER was mainly increased by the wash fraction whereas GM-CSF release was mainly induced by the flow through fraction. Further analysis by mass spectrometry failed to define the molecular species responsible for these effects.

### MAPK signaling pathways are involved in the regulation of barrier functions

**Figure 7.**
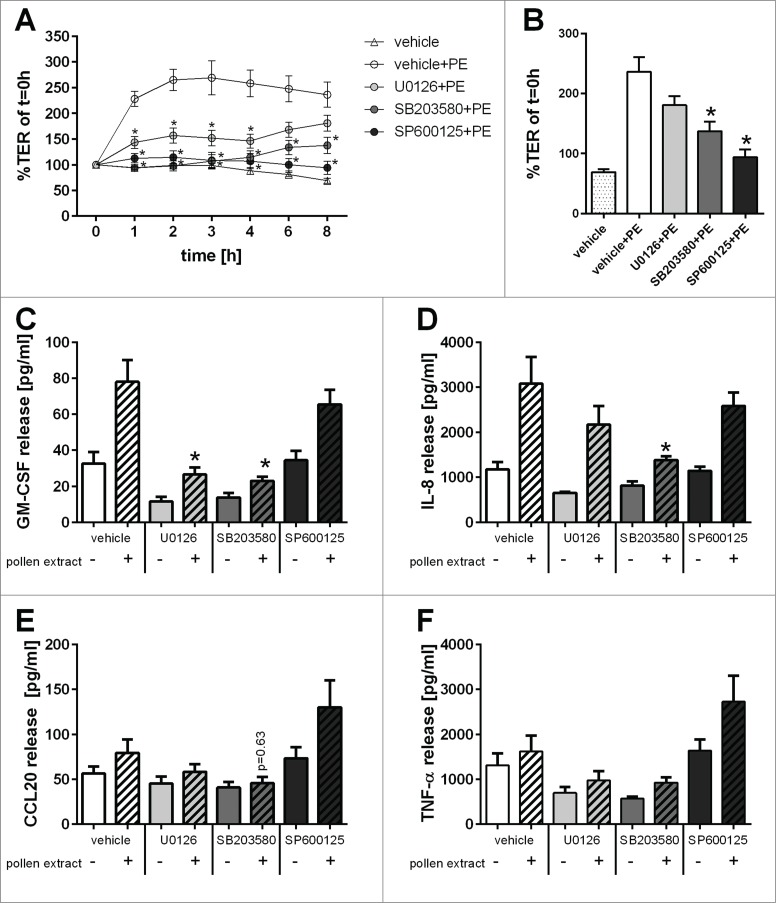
Involvement of MAPK signaling pathways in the regulation of bronchial epithelial barrier functions. Polarized 16HBEs were pre-treated for 30min with MAPK specific inhibitors (UO126: ERK1/2; SB203580: p38; SP600125: JNK) and subsequently exposed to an equivalent of 5mg/ml pollen. (**A**) Trans-epithelial resistance (TER) was measured over time after pollen exposure and normalized to t = 0h (n = 5). (**B**) TER at time point of 8h (n = 5). After 24h of exposure release of apical GM-CSF (**C**), apical IL-8 (**D**), basolateral CCL20 (**E**) and apical TNF-α (**F**) was analyzed by ELISA (n = 4–5). Mean±SEM; *: p ≤ 0.05 compared to pollen treated vehicle control (Mann-Whitney).

As our data implied differential regulation of the barrier responses to pollen extracts, we hypothesized that different intracellular signaling mechanisms would be involved in these responses. Since MAPK signaling pathways are among the main signaling pathways involved in regulation of epithelial barrier properties, we analyzed the effect of specific pharmacological inhibitors of these pathways on pollen extract mediated responses.^16,18^ As shown in **Figure 7A, B**, the increase in TER induced by pollen extract was completely abolished by the JNK inhibitor SP600125. The p38 inhibitor SB203580 was also a potent inhibitor of the pollen extract but its effect decreased over time and the TER started to recover. The ERK1/2 inhibitor U0126 showed a partial reduction in the pollen extract mediated TER increase, which only reached statistical significance in the first 4 h, but failed to reach statistical significance at 6 h and 8 h when the TER started to recover. In contrast, pollen-induced apical release of GM-CSF was completely inhibited by the ERK1/2 inhibitor U0126 and the p38 inhibitor SB203580 while the JNK inhibitor SP600125 had little effect (**Fig. 7C**). Pollen-induced apical release of IL-8 and CCL20 was reduced by the p38 inhibitor SB203580, while the ERK1/2 and JNK inhibitors were without effect (**Fig. 7D, E**). The apical release of TNF-α showed a trend for reduction with the ERK1/2 and p38 MAPK inhibitors, but this failed to reach statistical significance (**Fig. 7F**). In summary, pollen affected the epithelial ionic permeability barrier primarily through the JNK MAPK signaling pathway, whereas cytokine secretion was primarily mediated by the ERK1/2 and p38 signaling pathways.

### Adenosine receptor involvement in pollen-induced barrier modulation.

**Figure 8.**
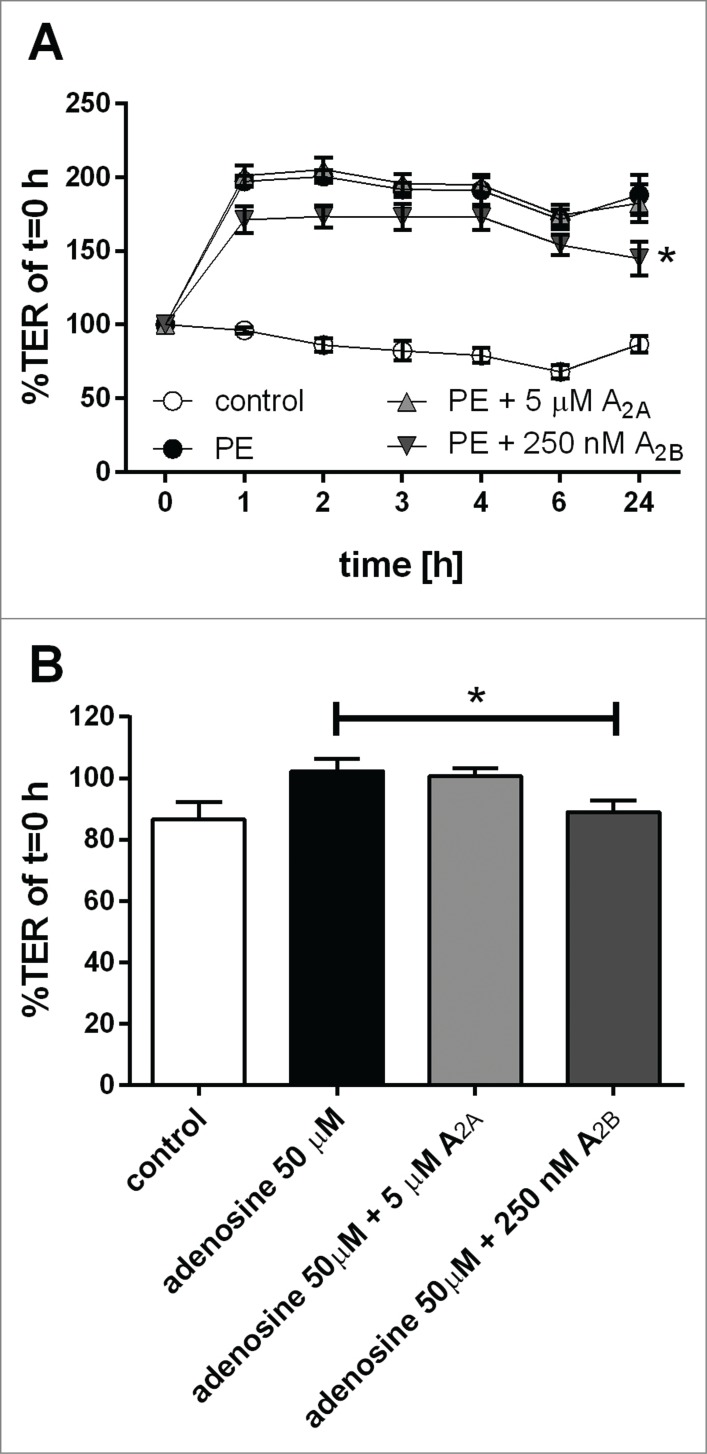
Effect of adenosine receptor antagonists on pollen-induced increase in physical barrier properties of airway epithelial cells. Polarized 16HBEs were pre-treated for 60 min with A_2A_ and A_2B_ adenosine receptor antagonists and subsequently exposed to pollen extract (PE) equivalent of 5 mg/ml pollen. Physical barrier properties were monitored by TER measurements over time (**A**). As control, adenosine and receptor antagonists were used and TER measurements after 24 h are shown (**B**). (n = 5); Mean ±SEM; *: p ≤ 0.05 compared to pollen or adenosine treatment (paired Student's t-test)

We previously identified the water-soluble nucleoside adenosine in grass pollen extract and showed that the function of dendritic cells is modulated by pollen-derived adenosine.^19^ Therefore, we tested if the A_2A_ and A_2B_ adenosine receptors were involved in pollen-mediated changes of bronchial epithelial barrier function. As shown in **Figure 8**, the A_2B_ receptor antagonist caused a small reduction of the pollen-mediated increase in TER while the A_2A_ receptor antagonist had no effect. Consistent with this, addition of adenosine to epithelial cultures caused a small increase in TER which was also blocked by the A_2B_ receptor antagonist. However, the pollen-induced release of IL-8 and TNF-α was not affected by adenosine receptor antagonists (**Fig. S6**). These data indicate that adenosine present in grass pollen may contribute to the pollen-mediated improved physical barrier in airway epithelial cells. However, other yet unidentified substances are also involved and regulate the physical and immunological barrier properties of airway epithelial cells.

## Discussion

In this study we found that exposure of bronchial epithelial cells to soluble components derived from pollens of grass, birch, ragweed, mugwort or pine resulted in a decrease in ionic permeability (i.e. increase in TER) of the epithelial barrier, even though protease activity was detectable in the extracts. Additionally, all pollen species induced polarized release of inflammatory mediators by the epithelium. Of the pollens tested, grass pollen derived substances showed the highest potential to affect bronchial epithelial barrier functions. Using ultrafiltration we demonstrated that low molecular weight substances below <3kDa were responsible for altered barrier functions in bronchial epithelial cells. Using ultra-performance liquid chromatography and mass spectrometry, we have previously shown that fraction comprises thousands of substances.^19^ Through use of reverse phase chromatography we determined that there are likely multiple components that affect the epithelium as the fraction that was most potent for stimulation of mediator release was ineffective in regard to decreasing ionic permeability. Consistent with this, the effects of the grass pollen extract on ionic permeability and cytokine release appeared to be due to differential activation of several pathways. JNK inhibitors markedly suppressed the change in ionic permeability over the duration of the experiment, whereas the p38 and ERK1/2 pathways appeared to contribute only to the early response to grass pollen substances. In contrast, release of GM-CSF and IL-8 was affected by ERK1/2 and p38 pathway inhibitors with relatively little contribution of JNK to these responses. Together, these data suggest that the physical and immunological barrier properties of the bronchial epithelium are differentially regulated.

Involvement of the JNK pathway in the regulation of epithelial cellular junctions has been shown previously, although effects are cell type, stimulus and duration-specific.^20^ For example, in intestinal epithelial cells JNK mediated disassembly of junctions via re-organization of the cytoskeleton and formation of contractile actin rings after calcium depletion.^21^ In contrast, in human bronchial epithelial cells JNK mediated an increase in transepithelial resistance after activation of the epidermal growth factor receptor (EGFR) and upstream Rac1 signaling pathways.^22^ Furthermore, it has been shown that activation of the JNK pathway increased the expression of tricellulin, a tight junction protein located at tricellular junctions, and increased physical barrier properties in epithelial cells.^23^ MAPK signaling pathways also regulate the release of inflammatory mediators like IL-8. While activation of the ERK pathway is thought to slightly increase IL-8 gene expression, activation of the p38 pathway stabilizes the mRNA resulting in increased protein expression.^24^ In this study, using the 16HBE cell line model we showed the involvement of p38 MAPK signaling pathways in the regulation of apical IL-8 release, which confirms our previous data using primary differentiated bronchial epithelial cells.^16^

Using fully differentiated human PBECs, we previously showed that the TER of differentiated PBECs was increased in the presence of pollen derived substances.^16^ Since we observed the same effect of pollen on the physical barrier properties in this study, the bronchial epithelial cell line 16HBEs was considered to be an appropriate model to analyze the effect of pollen substances on the barrier properties in more detail. Additionally, in our cell line model the pollen-induced changes in mediator release corresponded to the differentiated human PBECs model, with both models showing increased apical and basolateral release of IL-8 and just basolateral release of CCL20 in response to pollen.

After inhalation, pollen grains are hydrated on the airway mucosa and they release a multitude of substances in a short period of time; these substances range from proteins like allergens and proteases, to carbohydrates and lipid mediators.^11^ There is evidence that proteases released by pollen are able to disrupt the physical barrier of the epithelium.^25,26^ However, these studies used rather high pollen concentrations. In our model, the pollen concentrations are closer to natural exposure situations during the pollen season and despite detectable protease activity in the pollen extracts we did not observe any disruption of the physical barrier. Moreover, the increase in TER was mediated by low molecular weight substances and their effect may dominate over the effects of proteases. Potential candidate compounds which are able to alter airway epithelial barrier functions are pollen-associated lipid mediators, like PPE_1_.^11^ Previously, it has been shown that PPE_1_ is able to modulate dendritic cell function favoring a TH_2_ mediated immune response.^17^ In this study we showed that PPE_1_ did not significantly affect either cytokine release or the physical barrier of bronchial epithelial cells. Furthermore, although the endogenous lipid mediator lipoxin A_4_ has been shown to modulate airway epithelial barrier functions by increasing the physical barrier properties,^13^ we could not confirm this effect on TER, or any effect on cytokine release.

Epithelial barrier functions can be regulated by PPARγ, a member of the nuclear hormone receptor family and a receptor for many lipid mediators like prostaglandins.^14^ It has been shown that PPARγ agonists improve the physical barrier properties of nasal epithelial cells by inducing expression of tight junction proteins.^15^ Additionally, activation of PPARγ is associated with an anti-inflammatory role in the lung.^27–29^ Furthermore, it has been shown that the LPS-induced release of IL-12 by dendritic cells is inhibited by pollen-derived PPE_1_ via a PPARγ-dependent mechanism to support TH_2_ polarization.^17^ In the current study, we showed that PPARγ activation makes only a limited contribution to the pollen-induced increase in TER, since the PPARγ antagonist T0070907 had only a small suppressive effect on the pollen-induced TER response, and the PPARγ agonist ciglitazone alone had no effect. Of note, the PPARγ antagonist caused an increased release of GM-CSF and IL-8 by bronchial epithelial cells in the absence or presence of pollen extract. This can be explained by the interaction of PPARγ and NF-kB, where reduced PPARγ activity results in increased NF-kB activity,^30^ with the effect of stimulating inflammatory cytokine release.

In addition to lipid mediators, pollen extract contains many flavonoids, polyphenolic molecules which are ubiquitously present in plants. Biological effects have been described for many flavonoids, ranging from anti-inflammatory, anti-oxidant to anti-microbial.^31^ Of relevance to the current study, the flavonoid quercetin has been reported to improve physical barrier functions in epithelial cells.^32,33^ In this study we tested the effect of isorhamnetin, a methylated metabolite of quercitin identified in *Phleum pratense* pollen,^34^ onto the epithelial barrier properties. While the physical barrier properties were unaffected, isorhamnetin induced the release of GM-CSF and IL-8 to a level that was comparable to pollen extract. Since our results suggest, that the physical and immunological barrier properties are regulated by different mechanisms and substances, flavonoids present in pollen extract are a strong candidate for the activation of the immunological barrier.

Separation of the low molecular weight fraction of grass pollen extract by reverse phase chromatography showed that substances with a hydrophilic nature are responsible for the observed effects on epithelial barrier functions. In addition to flavonoids, potential candidate substances with a low molecular weight are purine and pyrimidine nucleosides. Adenosine, a purine nucleoside identified in grass pollen, has been shown to have immune-modulatory function on dendritic cells and thus influence T cell polarization.^19^ Using endothelial cells, a barrier improving effect of adenosine has previously been show.^35^ In the current study we showed that the adenosine A_2B_ receptor antagonist partially reduced the pollen induced increase in physical barrier functions indicating that adenosine may be one of many substances in pollen extract that influence the physical barrier properties of airway epithelial cells. However, other yet unidentified substances might be more effective or interact with each other to affect the epithelial barrier.

In summary we have shown that a variety of hydrophilic pollen substances, many yet to be identified, are able to differentially modulate the physical or immunological barrier properties of bronchial epithelial cells. Potential candidates that contribute to the overall effect of pollen extract on the airway barrier functions include the flavonoid isorhamnetin which modulates the immunological barrier and adenosine which affects the physical barrier. By using high performance liquid chromatography to further separate the active compounds it may be possible to identify specific components with distinct effects on epithelial cells rather than the mixed effect observed with the total pollen extract. Identification of specific substances in pollen altering the physical or immunological barrier of bronchial epithelial cells should increase our understanding of the regulatory mechanisms of epithelial barrier functions. Furthermore, identification of natural products that improve the physical barrier function might potentially be of benefit in the therapy of airway diseases where the physical barrier is dysfunctional.

## Material and Methods

### Cell culture

The human bronchial epithelial cell line, 16HBE14o- (a gift from Prof. DC. Gruenert, San Francisco, USA), was maintained in minimum essential medium (MEM) with Glutamax and supplemented with 10% foetal bovine serum and penicillin/streptomycin (Life technologies) (growth medium); cell culture flasks were coated with PureCol collagen I (Advanced BioMatrix). For experiment, 16HBE cells were cultured in collagen coated Transwell® permeable supports (diameter 6.5 mm, polyester membrane with 0.4 µm pores, Corning Life Sciences). Cells were seeded at a density of 1.5 × 10^5^ cells in 200 µl growth medium; the basolateral compartment contained 500 µl of the same medium. Medium exchange was carried out every 2–3 days. After 6 days, growth medium was exchanged and the apical medium was replaced by MEM without supplements. On day 7 the transepithelial resistance (TER) was measured using a EVOM voltohmmeter (World Precision Instruments); experiments were carried out when the cultures were polarized, having TERs higher than 1000 Ω.

### Aqueous pollen extracts (APE)

Pollen from Timothy grass (*Phleum pratense*), Ragweed (*Ambrosia artemisifolia*), Mugwort (*Artemisia vulgaris*), Birch (*Betula alba*) and Pine (*Pinus sylvestris*) were obtained from Allergon. Extracts of pollen were prepared by resuspending pollen grains in MEM without supplements at 30 mg/ml. After incubation at 37°C with agitation for 30 min, extracts were centrifuged, sterile filtered and stored in aliquots at −80°C until use.

### Stimulation of cells

Polarized 16HBE cells were stimulated apically with pollen extract. Concentrations cited in the results refer to the weight of pollen grains per ml before the extraction process. Directly after stimulation, TER was measured and monitored over time. TER measurements were normalized against the TER directly after stimulation (t = 0h). After 24 h, apical and basolateral supernatants were harvested and clarified by centrifugation; mediator release was analyzed by ELISA (GM-CSF ELISA: eBioscience; CXCL8/IL-8, CCL20 and TNF-α DuoSet ELISA kits: R&D Systems). Involvement of mitogen-activated protein kinase (MAPK) signaling pathways in the regulation of 16HBE barrier functions was investigated by using specific pharmacological inhibitors. U0126 was used as an inhibitor of the ERK1/2 pathway, SB203580 for blocking the p38 pathway and SP600125 for inhibiting the JNK pathway (all inhibitors from Sigma). Cells were treated apically with 25 µM of each inhibitor 30min before and during APE stimulation. DMSO was used as a vehicle control. Where tested, PPE_1_ (kindly provided by Prof. Martin J. Müller, University of Würzburg, Germany), lipoxin A4 (Enzo Life Sciences), isorhamnetin (Sigma) and adenosine (Sigma) were diluted in MEM and applied apically. T0070907 was used as a PPARγ antagonist and ciglitazone as PPARγ agonist (Cayman Chemical). SCH-442416 and MRS-1754 (Sigma) were used as an adenosine A2A and A2B receptor antagonist, respectively.

### Fluorescence microscopy

After 24 h of stimulation cells were fixed with 4% paraformaldehyde, permeabilised with 0.1% Triton X-100, blocked with 1% BSA in PBS and triple stained with an AlexaFluor647 labeled mouse monoclonal anti-ZO-1 antibody (clone ZO1-1A12), phalloidin-AlexaFluor488 and SytoxOrange (all from Life technologies). Stained membranes were mounted on slides using ProLong Gold antifade reagent (Life technologies) and analyzed with a LSM6000 microscope (Leica Microsystems). On z-stacks, a deconvolution was performed using Leica Application Suite software and orthogonal views were performed using ImageJ software.

### Isolation and characterization of pollen derived substances

Timothy grass pollen extract was fractionated by ultrafiltration using centrifugal filter units with a molecular weight cut-off of 3kDa (Millipore). Low molecular weight substances of the <3kDa fraction were further separated by reverse phase chromatography using BondElut Plexa cartridges (Agilent Technologies). Briefly, the <3kDa fraction was made alkaline with 2% NH_4_OH, applied to the conditioned cartridge and the flow through collected. After washing the cartridge with 2% NH_4_OH, bound substances were eluted with 100% methanol and isopropanol. Each fraction was collected and lyophilized for mass spectrometry (see below) and assessment of bioactivity. In the latter case, the fractions were redissolved in half the volume of the original extract in MEM to account for the loss during the separation process and used for cell stimulation.
