# Supplementary material for: Low molecular weight components of pollen alter bronchial epithelial barrier functions
Source: Tissue Barriers. 2015 Jul 15;3(3):e1062316. doi: 10.1080/15476286.2015.1062316 (PMC4574901; doi:10.1080/15476286.2015.1062316)
Supplement: Supplemental_Files.zip [file ktib-03-03-1062316-s001.zip › Supplemental Files/Supplemental Figure Captions.docx]

**Supplemental Figure 1.** Protease activity in pollen extract of different species. Using a fluorescence protease assay, the activity of proteases in pollen extract equivalent of 30 mg/ml was detected (n=3). Mean ±SEM.

**Supplemental Figure 2.** The physical barrier functions of bronchial epithelial cells are not affected by phytoprostane E1 (PPE_1_) or lipoxin A4 (**A and B**). The PPARγ antagonist T0070907 caused only a partial reduction of pollen induced increase in TER in the first 2-3 h, while the PPARγ agonist ciglitazone did not cause an increase in TER (**C**). Polarized 16HBEs were stimulated apically with PPE_1_, lipoxin A4, ciglitazone or pollen extract (PE) equivalent to 5 mg/ml. Cells were pre-stimulated with T0070907 prior exposure to pollen. Trans-epithelial resistance (TER) was monitored over time and normalized to t=0 h. (**A**: n=5; **B** **and** **C**: n=4); Mean ±SEM.

**Supplemental Figure 3.** Phytoprostane E1 (PPE_1_) alters the immunological barrier function of the bronchial epithelium. Polarized 16HBEs were apically stimulated with PPE_1_ or grass pollen extract (PE) equivalent of 5 mg/ml pollen for 24 h. The release of GM-CSF (**A**), IL-8 (**B**) and TNF-α (**C**) were analyzed by ELISA (n=4-5). Mean ±SEM; *: p≤0.05 compared to control (Mann-Whitney).

**Supplemental Figure 4.** Effect of lipoxin A_4_ and involvement of PPARγ on the immunological barrier functions of bronchial epithelial cells. Polarized 16HBE cells were apically exposed to grass pollen extract (PE, equivalent of 5 mg/ml pollen), lipoxin A_4_ and PPARγ antagonists T0070907 in combination with PE and PPARγ agonist ciglitazone. After 24 h of exposure release of GM-CSF (**A**) and IL-8 (**B**) was analyzed by ELISA. (n=3), Mean ±SEM; *: p≤0.05 compared to control (Wilcoxon).

**Supplemental Figure 5.** Effect of the flavonoid isorhamnetin on epithelial barrier properties. Polarized 16HBEs were apically stimulated with 50 μM isorhamnetin and the low molecular weight fraction of pollen extract (PE<3kDa) equivalent to 5 mg/ml pollen. Physical barrier properties were monitored by TER measurements over time (**A**). After 24 h of stimulation apical release of GM-CSF and IL-8 (**B**) was analyzed by ELISA. (n=4), Mean ±SEM; *: p≤0.05 compared to untreated control (paired Student’s t-test).

**Supplemental Figure 6.** Effect of the adenosine receptor antagonists on pollen-induced release of IL-8 and TNF-α by airway epithelial cells. Polarized 16HBEs were apically pre-stimulated with A_2A_ or A_2B_ adenosine receptor antagonists for 60 min and subsequently exposed to pollen extract equivalent to 5mg/ml pollen. After 24 h of stimulation the release of IL-8 (**A**) and TNF-α (**B**) was analyzed by ELISA in the apical and basolateral supernatants. (n=5); Mean ±SEM.
