# Supplementary figures and images for: Low molecular weight components of pollen alter bronchial epithelial barrier functions
Source: Tissue Barriers. 2015 Jul 15;3(3):e1062316. doi: 10.1080/15476286.2015.1062316 (PMC4574901; doi:10.1080/15476286.2015.1062316)

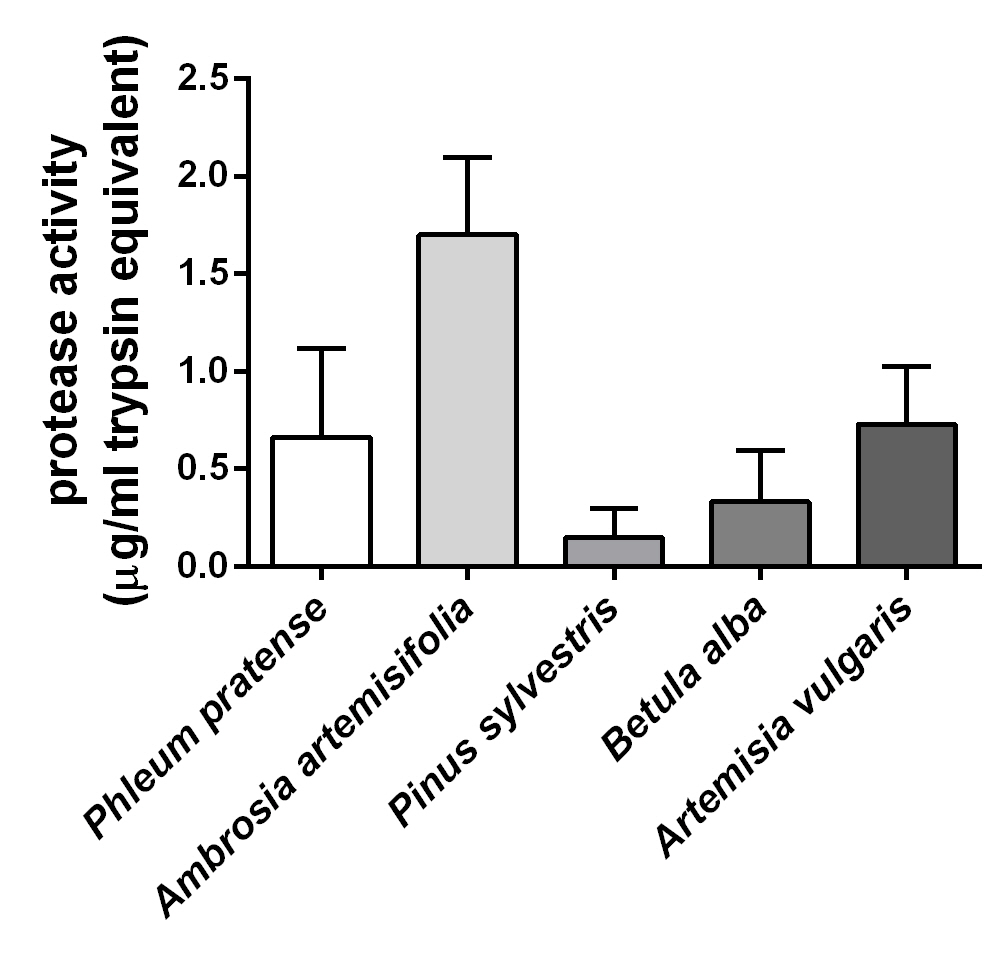

Supplement: Supplemental_Files.zip [file ktib-03-03-1062316-s001.zip › Supplemental Files/Supplemental Figure 1.jpg]

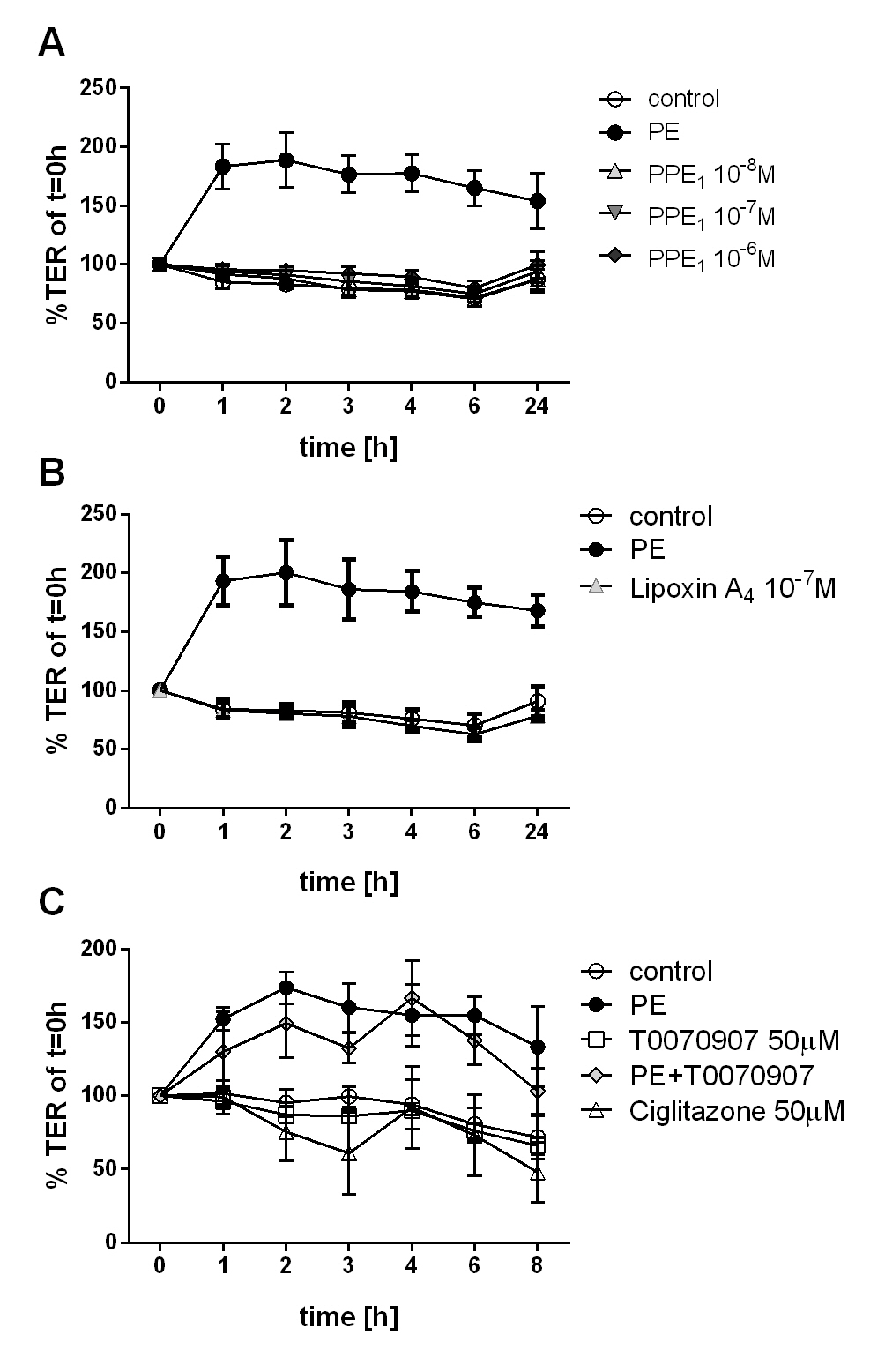

Supplement: Supplemental_Files.zip [file ktib-03-03-1062316-s001.zip › Supplemental Files/Supplemental Figure 2.jpg]

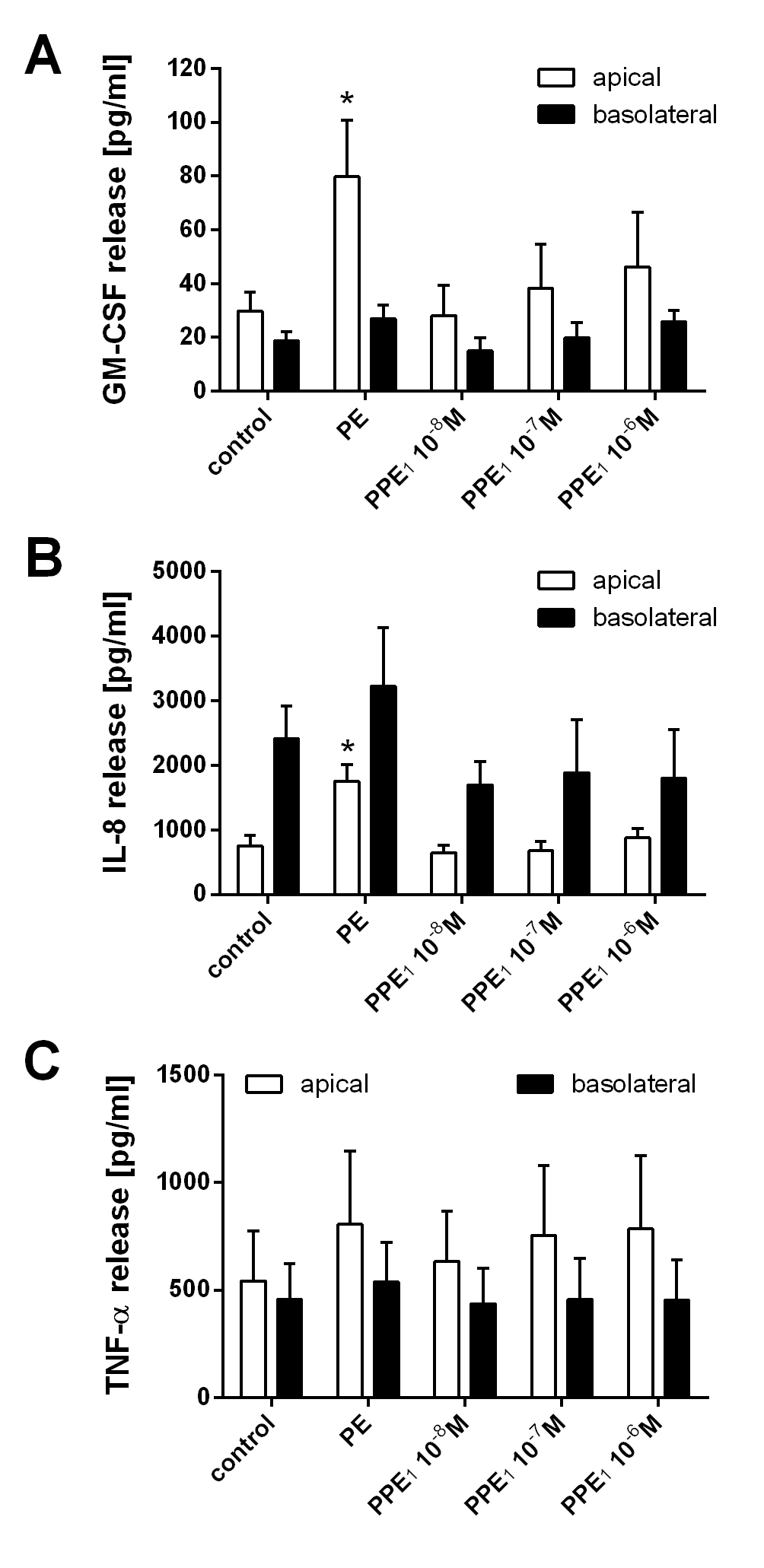

Supplement: Supplemental_Files.zip [file ktib-03-03-1062316-s001.zip › Supplemental Files/Supplemental Figure 3.jpg]

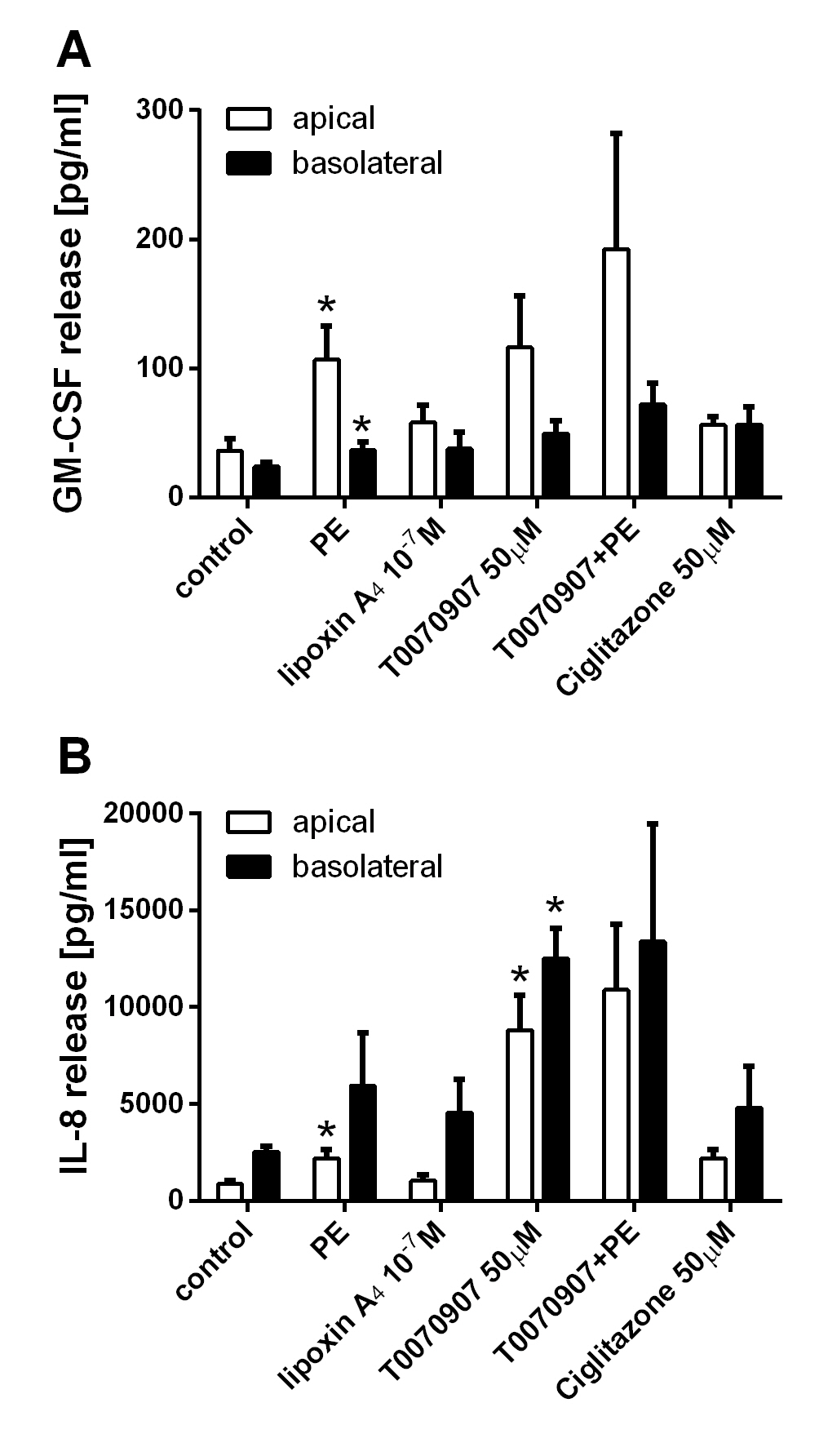

Supplement: Supplemental_Files.zip [file ktib-03-03-1062316-s001.zip › Supplemental Files/Supplemental Figure 4.jpg]

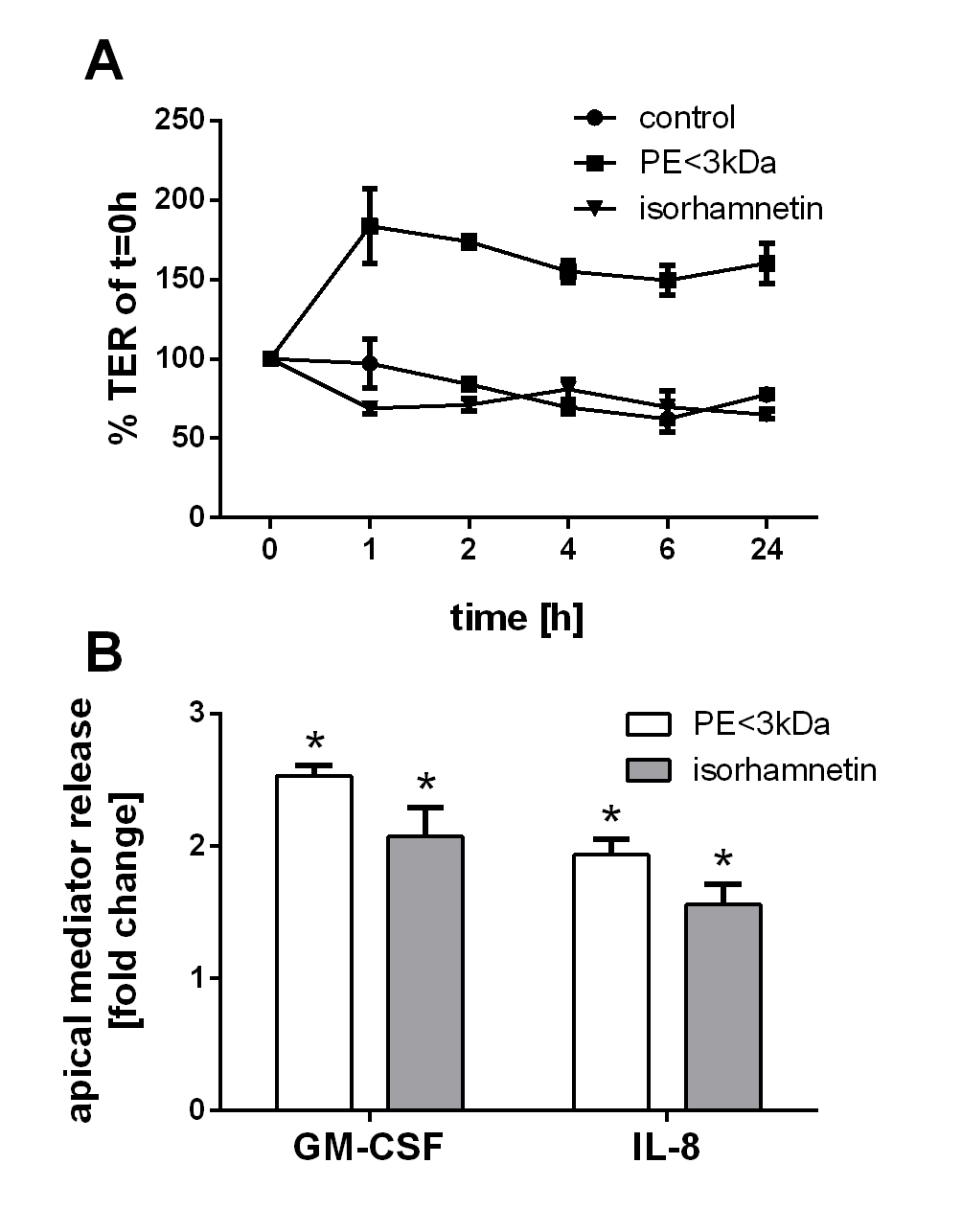

Supplement: Supplemental_Files.zip [file ktib-03-03-1062316-s001.zip › Supplemental Files/Supplemental Figure 5.jpg]
